# Large tumoral pseudoangiomatous stromal hyperplasia with ER/PR stromal negativity in a 20‐year‐old female: A rare case report

**DOI:** 10.1002/ccr3.8398

**Published:** 2024-01-02

**Authors:** Somar Mansour, Seif‐Aldin Abdul Rahman, Ali Kazour, Ibrahim Salama, Hussain Shmayes, Samer Rajab, Rana Issa

**Affiliations:** ^1^ Department of Pathology Cancer Research Center, Tishreen University Hospital Latakia Syria; ^2^ Department of Obstetrics and Gynecology Cancer Research Center, Tishreen University Hospital Latakia Syria; ^3^ Faculty of Medicine Tishreen University Latakia Syria; ^4^ Department of Internal Medicine Tishreen University Hospital Latakia Syria; ^5^ Department of General and Thoracic Surgery Tishreen University Hospital Latakia Syria

**Keywords:** breast tumor, case report, gigantomastia, pseudoangiomatous, stromal hyperplasia

## Abstract

Pseudoangiomatous stromal hyperplasia (PASH) is a rare lesion of the breast stromal tissue with unknown mechanism. Hormonal stimulation of mammary myofibroblasts is the most important theory due to stromal positivity of progesterone receptor (PR) or/and estrogen receptor (ER). We report a case of PASH with stromal PR/ER negativity.

## 1 INTRODUCTION

Pseudoangiomatous stromal hyperplasia (PASH) is a rare, proliferative, benign breast lesion that was first described in 1986 by Raza et al.^1^

It most commonly occurs during the premenopausal period and is often found incidentally in breast biopsy, but it can occasionally grow in some cases to form an extremely large mass.^2^ It has a physical and psychological effects on the patient's health. The etiology is poorly understood but studies showed that hormonal changes could be the main factor because of the positivity of progesterone receptor (PR) and/or estrogen receptors (ER) in most cases. The treatment depends on the size of the mass and surgical procedure is the gold standard treatment in large symptomatic cases.^3^

Herein, we report a rare case of PASH with negative stromal PR/ER expression and was developed during adolescence.

## 2 PRESENTATION

A 20‐year‐old female presented to the Obstetrics and Gynecology clinic with a complaint of painless enlargement in her left breast. The lump has gradually increased in size over the last 5 years and was associated with back pain. She had no family history of breast or ovarian cancer.

Physical examination revealed a large solid mass with a well‐circumscribed margin in the left breast and two small palpable masses in the right breast. There was no nipple discharge, retraction or skin changes and no palpable lymph nodes were noticed.

Hormonal laboratory tests were ordered (beta human chorionic gonadotropin, thyroid‐stimulating hormone, luteinizing hormone, follicle‐stimulating hormone, prolactin, liver function tests) and were within the normal range.

An ultrasound examination was performed showing a heterogeneous, hypoechoic large mass within the left breast tissue, with circumscribed margins and no significant vascularity involving the lateral aspect of the left breast and based on these findings, the lesion was categorized as breast imaging reporting and data system (BIRADS) III/3. In addition, two hypoechoic well‐defined lesions were noticed within the right breast tissue.

**FIGURE 1:**
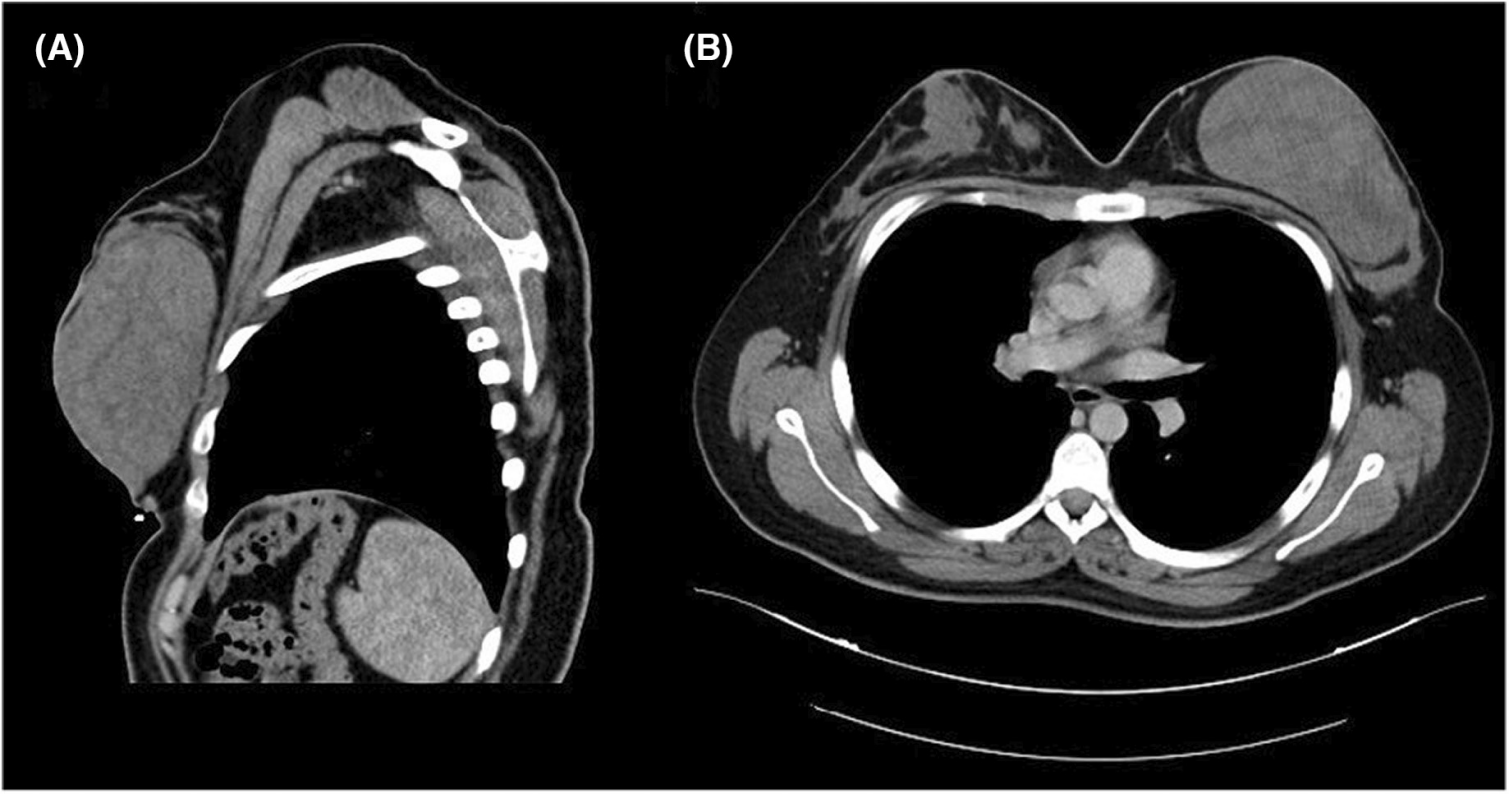
(A) Sagittal CT scan showing a large well‐circumscribed rounded mass measuring (13 × 8) cm occupying most of the left breast; (B) Axial CT section showing two small well‐defined nodular lesions in the right breast with a large mass in the left breast.

Furthermore, a CT scan was carried out and showed a well‐circumscribed rounded mass that measures 13 × 8 cm, occupying most of the left breast (Figure 1).

**FIGURE 2:**
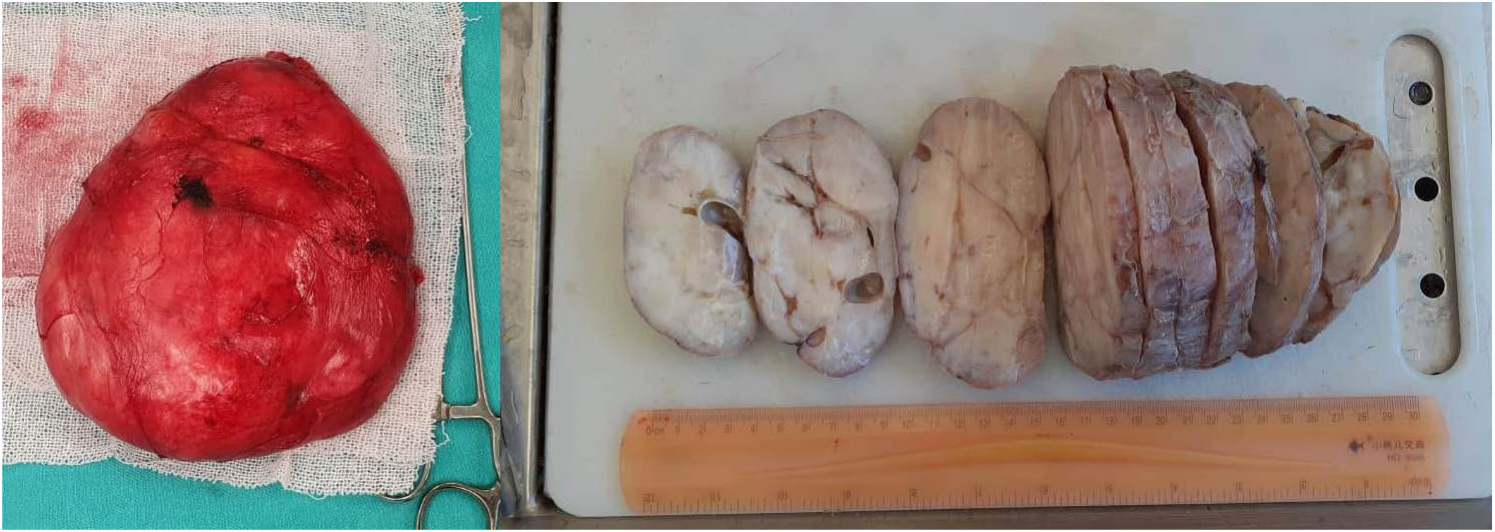
Gross inspection of the left breast mass.

**FIGURE 3:**
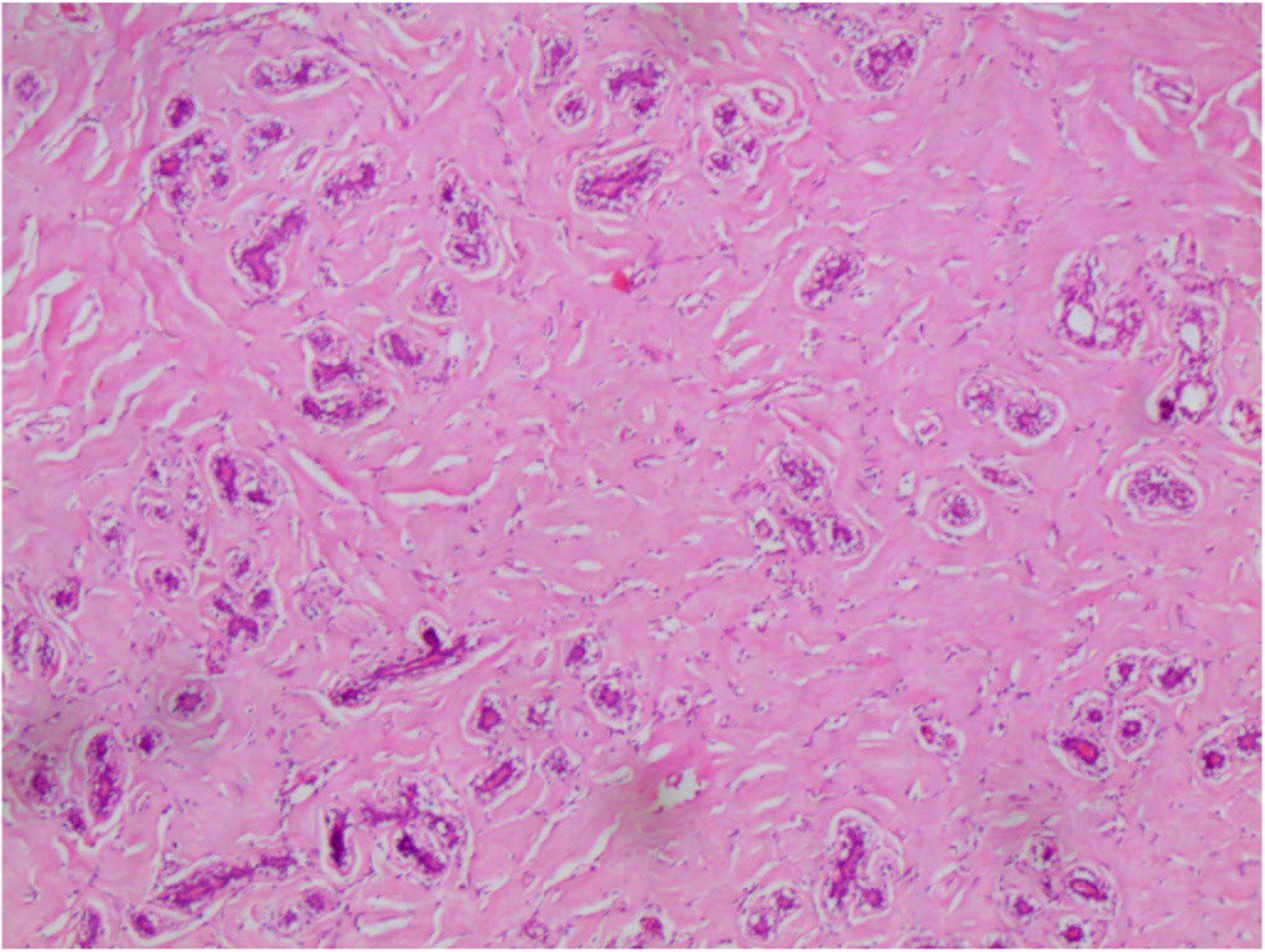
Terminal duct lobular units with intervening collagenous stroma showing vascular‐like anastomosing empty spaces (hematoxylin and eosin stain original magnification 200×).

In addition, well‐defined masses in the right breast were detected with the largest one measuring (2 cm). Based on these findings, a differential diagnosis of benign tumors such as atypical fibroadenoma or phyllodes tumors was suspected. Given the size of the tumor, the surgeon recommended and performed a surgical excision of the lesions by making an inferior circumareolar incision. The gross inspection of the specimens revealed three well‐circumscribed firm white‐gray masses (Figure 2). Pathological assessment of the largest mass tissue revealed normally structured terminal duct lobular units with occasional cystic dilated ducts lined by apocrine metaplastic cells. The intervening dense collagenous stroma contained vascular‐like anastomosing empty spaces lined by spindle cells (Figure 3). There was no evidence of necrosis or atypia.

**FIGURE 4:**
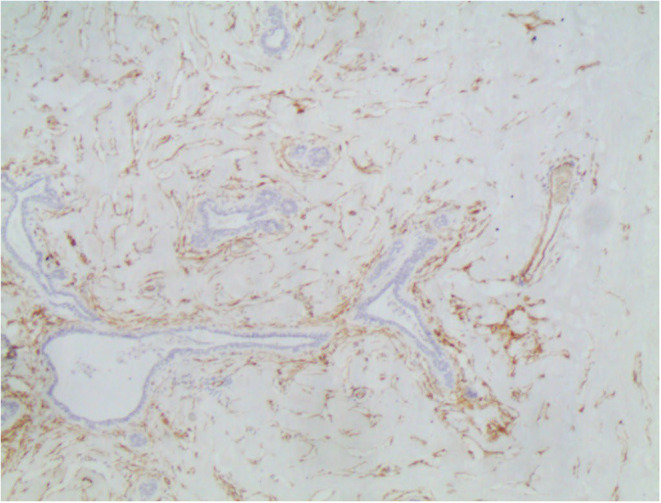
Immunohistochemistry showing positivity for CD34 in the stromal spindle cells.

**FIGURE 5:**
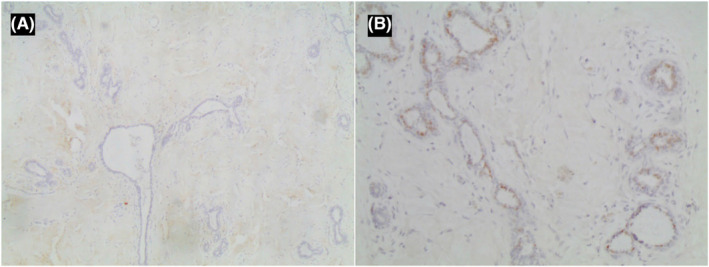
(A) Immunohistochemistry showing estrogen receptor negativity in the epithelial and stromal component; (B) Immunohistochemistry showing PR negativity in the stromal component and positivity in the epithelial component.

Subsequent immunohistochemistry was positive for CD34 and negative for CD31 (Figure 4). In addition, ER was negative in the epithelial and stromal components (Figure 5A), while PR was negative in the stromal component and positive in the epithelial component (Figure 5B). The diagnosis of tumoral (PASH) was reported for the left breast mass. The other lesions were diagnosed as fibroadenomas.

## 3 DISCUSSION

Tumoral PASH is a rare benign lesion that is characterized by a dense, collagen‐rich proliferation of mammary stroma, forming inter‐anastomosing capillary‐like spaces. It occurs mostly in premenopausal and less commonly in postmenopausal women who are on hormone replacement therapy.^4^

The etiology and pathogenesis are not fully elucidated, but they may be linked to endogenous hormone stimulation of mammary myofibroblasts. This hypothesis is supported by the expression of progesterone receptors on the nuclei of myofibroblasts in PASH lesions and the high prevalence of premenopausal women among patients with PASH. Other theories include that PASH is an independent myofibroblastic lesion of the breast or an abnormal proliferation of lymphatic channels.^5^

Most of the cases occur between 30 and 50 years and they are often found incidentally in breast biopsy (23% of the 200 consecutive breast biopsy specimens in Ibrahim et al. study indicated the presence of PASH).^6^, ^7^

PASH can be found in the stroma of fibroadenomas or phyllodes tumors and rarely forms a mass as found in our case. In core needle biopsies, the diagnosis of tumoral PASH is a diagnostic challenge as it is—in most of cases—reported with other pathological entities. Fibroadenomas and phyllodes tumors proliferation involve both stromal and glandular elements, whereas PASH tumor is purely a stromal proliferation.^8^

Although the prevalence of PASH by age varies by studies, it is uncommon to occur in females during adolescence as found in our case.

Clinically, it is often asymptomatic and symptomatic cases usually present with a painless palpable mass, gradually increasing in size. The size of the masses is reported to be between 1 cm and 18 cm.^9^

Unfortunately, PASH does not exhibit any distinctive radiological characteristics and can mimic many entities such as fibroadenomas or hamartomas. It appears on ultrasound as an oval or round homogeneous well‐circumscribed mass.^4^

Establishing the diagnosis is based on histopathological and immunohistochemical evaluation. PASH typically shows stromal cell proliferation and over‐secretion of collagen, with slit‐like spaces lined by spindle cells (myofibroblasts). Myofibroblasts proliferation and collagen over‐secretion create a solid tissue with cystic area that resemble ectatic vessels (pseudovascular spaces).^6^ The spindle cells are positive for Vimentin, CD34, BCL2, CD99, and *α*‐smooth muscle actin but negative for CD31 and factor VIII (an endothelium‐specific marker). In addition, the spindle cells in the stroma are hormonally sensitive and frequently express PR and less frequently ER.^10^

According to Erin Bowman et al study, 95% of the PASH tumors stained positive for ER and/or PR receptors. In our case, both receptors were negative in the stromal component which is one of the factors that makes our study unique.^11^

For the management of PASH, surgical excision is the preferred treatment choice. However, the “watch‐and‐wait” strategy can be considered if the size of the lump is smaller than 2 cm or the diagnosis of PASH is made on core biopsy. The recurrence rate after surgery is reported in range from 9% to 21%.^10^

## 4 CONCLUSION

Tumoral PASH is a rare lesion of the breast stromal tissue with an excellent prognosis. Regardless of its rarity, PASH should be considered an important lesion in the differential diagnosis of breast enlargement cases. In this case report, we reported an uncommon case of large nodular PASH that was developed during adolescence. In addition, our article highlights the diagnostic challenges of this rare tumor‐like entity and emphasizes the importance of histologic and immunohistochemical assessment in accurate diagnosis, as PASH, clinically and radiologically, can closely mimic many entities.

## AUTHOR CONTRIBUTIONS

**Somar Mansour:** Conceptualization; writing – original draft. **Seif‐Aldin Abdul Rahman:** Conceptualization; writing – original draft. **Ali Kazour:** Writing – review and editing. **Ibrahim Salama:** Writing – review and editing. **Hussain Shmayes:** Writing – review and editing. **Samer Rajab:** Writing – review and editing. **Rana Issa:** Supervision; writing – review and editing.

## FUNDING INFORMATION

None.

## CONFLICT OF INTEREST STATEMENT

The authors declare that they have no competing interests.

## CONSENT

Written informed consent was obtained from the patient to publish this report in accordance with the journal's patient consent policy.

## Data Availability

The data that support the findings of this study are available from the corresponding author upon reasonable request.
